# Crystalline Characteristics, Mechanical Properties, Thermal Degradation Kinetics and Hydration Behavior of Biodegradable Fibers Melt-Spun from Polyoxymethylene/Poly(l-lactic acid) Blends

**DOI:** 10.3390/polym11111753

**Published:** 2019-10-25

**Authors:** Jianhua Li, Yatao Wang, Xiaodong Wang, Dezhen Wu

**Affiliations:** 1 State Key Laboratory of Organic–Inorganic Composites, Beijing University of Chemical Technology, Beijing 100029, China; ecljh@kailuan.com.cn; 2 Coal Chemical R & D Center, Kailuan Group Limited Liability Corporation, Tangshan 063018, China; wangyatao@kailuan.com.cn

**Keywords:** biodegradable bicomponent fibers, polyoxymethylene/poly(l-lactic acid) blends, melt spinning, post drawing, tensile properties, crystalline orientation, hydration behavior, thermal degradation kinetics

## Abstract

A series of polyoxymethylene (POM)/poly(l-lactic acid) (PLLA) blends were prepared by melt extrusion, and their spinnability was confirmed by rheological characterizations, successive self-nucleation, and annealing thermal fractionation analysis. The bicomponent fibers were prepared by means of the melt-spinning and post-drawing technologies using the above-obtained blends, and their morphology, crystalline orientation characteristics, mechanical performance, hydration behavior, and thermal degradation kinetics were studied extensively. The bicomponent fibers exhibited a uniform diameter distribution and compact texture at the ultimate draw ratio. Although the presence of PLLA reduced the crystallinity of the POM domain in the bicomponent fibers, the post-drawing process promoted the crystalline orientation of lamellar folded-chain crystallites due to the stress-induced crystallization effect and enhanced the crystallinity of the POM domain accordingly. As a result, the bicomponent fibers achieved the relatively high tensile strength of 791 MPa. The bicomponent fibers exhibited a partial hydration capability in both acid and alkali media and therefore could meet the requirement for serving as a type of biodegradable fibers. The introduction of PLLA slightly reduced the thermo-oxidative aging property and thermal stability of the bicomponent fibers. Such a combination of two polymers shortened the thermal lifetime of the bicomponent fibers, which could facilitate their natural degradation for ecological and sustainable applications.

## 1. Introduction

Polyoxymethylene (POM) has been broadly recognized as one of the most important engineering thermoplastics due to its prominent mechanical strength and impact toughness, outstanding anti-fatigue performance, excellent electrical insulation, good chemical stability, weathering resistance, and unique self-lubrication feature. By now, POM has gained a broad application in the fields of electrics, machinery, automobiles, construction, transportation, agriculture, medicine, sports and so on [1]. As a semi-crystalline polymer with a linear molecular chain based on the repeated –CH_2_–O– units, POM is commercially available in homo-polymerization and co-polymerization types and can easily be processed by injection-molding, blow-molding, and extrusion methods because of its thermoplastic nature [2]. Like most of the semicrystalline thermoplastic polymers with good fluidity, POM seems to be employed to prepare synthetic fibers by a melt-spinning method. This provides an impetus for the development of spinning techniques to produce the synthetic fibers with both high performance and reasonable price [3].

Although the melt-spun POM fibers were first reported in the early 1970s [4], there have been seldom successful cases claimed by now, which may be ascribed to a great difficulty in the melt-spinning processing technology of POM resin. As a matter of fact, POM resin possesses a rather high degree of crystallinity and a very fast crystallization rate, especially for the homo-polymerization-type resin. This results in enormous technical challenges in the post-drawing process for as-spun POM fiber [5,6]. It was found that both the folded-chain crystals and extended-chain crystals could grow in the cooling process of as-spun POM fibers [7], and the folded-chain crystallites could transform into the extended-chain ones during the post-drawing process for the as-spun fibers [8,9], and therefore a large number of microvoids were formed in the fibers, leading to the deterioration of mechanical properties of POM fibers [10]. In order to suppress the formation of microvoids, Komatsu et al. [11,12,13,14] developed a continuous drawing process for the melt-spun POM fiber under a pressure and found the pressure influenced the structural characteristics and mechanical properties of the resulting fibers significantly. Moreover, the combination of POM with a small number of other co-monomers has also been considered as an effective solution for the high degree of crystallinity of POM. As a result, the high-performance POM fibers could be obtained by controlling the crystallization of the as-spun fibers during the post-drawing process [15,16]. Many studies indicated that the copolymerization-type POM resin presented poorer crystallinity than the homopolymerization-type one, because there was 3–5 wt % of *1,3*-dioxolane randomly distributed in its macromolecule. These co-monomers can effectively reduce the degree of crystallinity and crystallization rate of POM [17]. However, the copolymerization-type POM resin is still not suitable for preparation of the POM fibers with satisfactory performance due to its poor melt-spinning processability. Therefore, the POM fibers are still not available as a commercialized product in the worldwide marketplace of synthetic fibers.

In our previous study, we made a breakthrough in the preparation technology of POM fibers by using a commercial injection molding-grade POM resin through the melt-spinning and post-drawing processes, with a high modulus of 50.2 cN/dtex (6.9 GPa), high tensile strength of 6.7 cN/dtex (925 MPa), and moderate elongation of 15.6% achieved for the resulting fiber [18]. Such a type of POM fiber exhibits a great potential in the application for reinforcement of inorganic building materials such as cements, concretes, plasters, gypsum, and geopolymers [19]. Although POM fibers can well meet the requirement for these highly demanded applications, they are not biodegradable because of the stable molecular structure of POM [20,21,22]. With rising environmental concerns and the focus on sustainable development as a worldwide tendency, there is growing awareness of the importance of biodegradable polymeric materials and their products in our ordinary daily life [23]. It is highly desirable that partially biodegradable performance can be achieved for POM fibers from the blends of POM resin with other biodegradable polymers, which can impart POM fibers with an ecologically friendly feature and makes them meet the requirement for sustainable development [24,25]. In recent years, the miscibility and crystallization characteristics of POM/poly(l-lactic acid) (PLLA) blends have been investigated extensively [26,27,28], and it has been found that POM is partially miscible with PLLA due to the fact that there is an interchain hydrogen bonding interaction between the two domains in the amorphous region [29]. Moreover, both POM and PLLA are semi-crystalline polymers, and have almost similar melting temperatures and melt flow rates [30]. This may facilitate the management of viscosity of the blends to obtain an optimum dispersion. Mathurosemontri et al. [31] found that a biodegradable thermoplastic material with improved mechanical properties could be obtained from the high-speed injection molding of POM/PLLA blends due to an enhancement of phase distribution of PLLA in the POM domain.

It is well known that the blend of two polymers is one of the effective ways to control the crystallization rate and degree of crystallinity, and furthermore it has been confirmed that polymer blending is one of the most important ways to develop a new biodegradable polymeric material through the blending of a biodegradable polymer with the non-degradable polymers [32,33,34]. Our previous study also demonstrated that the combination of POM with a small amount of PLLA could create a potentially biodegradable material with satisfactory mechanical properties and enzymatic degradation performance [35]. Therefore, it is possible to obtain a type of biodegradable synthetic fibers from POM/PLLA blends by a melt-spinning method on the basis of the technical breakthrough in the preparation of melt-spun POM fibers in our previous work [18]. In this work, we attempted to develop a novel type of biodegradable bicomponent fibers based on the POM/PLLA blends through melt spinning and post drawing. The preparation methodology, crystallization characteristics, orientation structure, mechanical performance, hydration behavior, and thermal degradation kinetics of the obtained bicomponent fibers were investigated extensively, and their lifetimes were predicted according to the data derived from the thermal degradation kinetic study. It is expected that the POM/PLLA bicomponent fibers developed by this study can achieve appropriate mechanical and biodegradable properties to meet the requirement for sustainable industrial and domestic applications.

## 2. Materials and Methods

### 2.1. Materials

The injection molding-grade copolymerization-type POM resin with a number-average molecular weight of above 30,000 was kindly supplied by Tangshan Zhonghao Chemical Co. Ltd., Kailuan Group, Tangshan, China. A commercial grade product of PLLA resin (commercial grade: 3052D) with a melt flow index of 14.0 g/10 min was purchased from Nature Works Co. LLC, Minnetonka, MN, USA. This PLA resin has a number average molecular weight of 72,300 g/mol and an l-lactide content of 96.5 wt %. The antioxidants (IRGANOX^®^ 245 and IRGAFOS^®^ 168) were purchased from BASF East Asia Regional Co., Ltd., Shanghai, China. Hydrochloric acid (HCl, 37.5 wt %) and sodium hydroxide (NaOH) were commercially obtained from Sinopharm Chemical Reagent Co., Ltd., Shanghai, China and used as received without further purification.

### 2.2. Preparation Methods

POM and PLLA pellets were fully dried in a vacuum oven at 80 °C for 12 h prior to use. The pellets of two polymers were premixed at the POM/PLLA mass ratios of 95/5, 90/10, and 80/20 with various processing additives in a high-speeding mixer and then melt blended by a ZSK Mc18 co-rotating twin-screw extruder (Coperion Nanjing Machinery Co., Ltd., Nanjing, China) with a screw diameter of 32 mm and an L/D ratio of 42:1. The rotation speed of screw was set as 150 rpm, and the temperatures were set as 170, 175, 190, 190, 185, and 185 °C from feeding zone to extruding die with a gradually increasing gradient. The extruded strands were quenched in a water bath and then pelletized into granules using a pelletizer. The obtained POM/PLLA blends were dried in a vacuum oven at 80 °C for 24 h to ensure enough low moisture absorption prior to the use for melt spinning.

**Figure 1:**
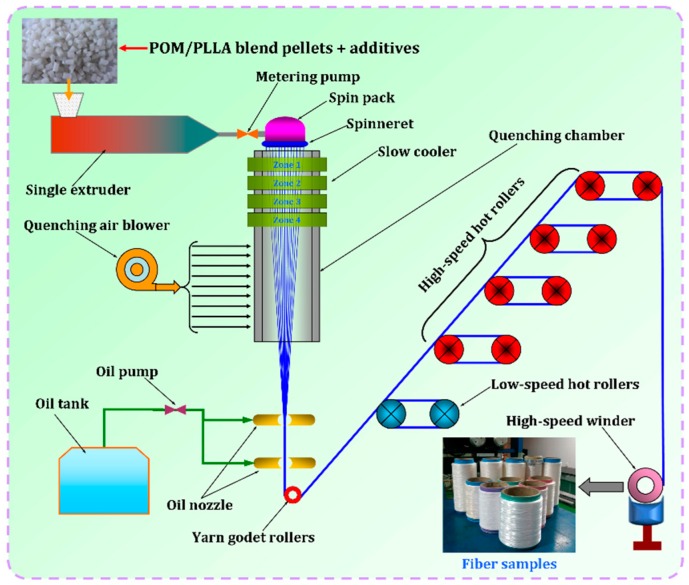
Schematic representation of melt-spinning and post-drawing processes for POM/PLLA bicomponent fibers.

The POM/PLLA bicomponent fibers were prepared via melt spinning and post drawing by means of a custom-designed melt-spinning device composed of a single-screw extruder with a screw diameter of 20 and an L/D ratio of 25/1, a melt transportation system equipped with a meter pump, a spin pack equipped with a spinneret (72 orifices, 0.3 mm), a quenching chamber equipped with a slow cooler followed by a cross air blower, a set of oil jetting installation equipped with a pair of oil nozzles, a yarn godet roller, a pair of low-speed drive rollers, four pairs of drive rollers, and a high-speed a pair of low-speed winder. Figure 1 shows the schematic representation of this device. The temperatures of the single-screw extruder were set to 180, 195, 200, and 205 °C along the barrel from feeding zone to die. The blend pellets were fully melted at these extrusion temperatures and then pushed into the spin pack at a temperature of 205 °C. Meanwhile, the melt pressure was controlled at around 12 MPa in the spin pack. After passing through spinneret orifices into the air, the melt flow was quenched in a slow cooling process through 4 temperature zones of 170, 165, 160, and 155 °C and then was cooled by the conventional cross air at about 120 °C. The solidified filaments were coated with oil and then drawn directly by a pair of low-speed hot rollers at 90 °C, followed by post-drawing by four pairs of high-speed hot rollers at 120 °C to obtain the final bicomponent fibers at different draw ratios under the control of the rotation speed of the high-speed hot rollers. Finally, the drawn fiber bundle was wound up by a high-speed yarn winder. This melt-spinning and post-drawing procedure can be vividly observed from a video in Supplementary Materials.

### 2.3. Characterizations and Measurements

The melt flow rates (MFRs) of blend samples were measured on a SANS ZRZ1452 melt flow indexer (MTS Systems China Co., Ltd., Shenzhen, China) according to the international standard of ISO 1133–2011. The samples were heated and extruded at 190 °C under a load of 2.16 kg through a capillary with a diameter of 2.095 mm for 10 min, and the data of MFR were reported as an average of five tests. The apparent melt viscosities of blend samples were measured with an SR20 capillary rheometer (Instron Corporation, Boston, MA, USA) with a capillary diameter of 1.262 mm and an L/D ratio of 10.51. The tests were carried out at 195, 205, and 215 °C at shear rates ranging from 1 to 5000 s^−1^. The differential scanning calorimetry (DSC) was performed to evaluate the melting behavior of blend samples on a Q20 differential scanning calorimeter (TA instruments, New Castle, UK) at a heating rate of 10 °C/min under a nitrogen flow of 20 mL/min. Before the formal measurement, the samples were heated up to 190 °C at a heating rate of 50 °C/min and held at this temperature for 5 min to erase the processing and heat histories. Moreover, considering that only the POM phase can crystallize during the cooling process, the degree of crystallinity (*X*_c_) of the bicomponent fibers should be normalized to the POM phase by its weight fraction and could be calculated by Equation (1):

(1) $$X_{c}=\frac{\Delta H_{m}}{(1-w)\cdot \Delta H_{m}^{o}}\times 100\%$$

where Δ*H*_m_ is the melting enthalpy of POM/PLLA blend fibers obtained from the DSC analysis result, *w* the weight fraction of PLLA, and Δ*H*_m_° refers to the melting enthalpy of pure POM in a 100% crystalline form, which is set as 326 J/g [36]. The successive self-nucleation and annealing (SSA) thermal fractionation of blend samples were also performed with the same DSC instrument under the nitrogen atmosphere. The samples were first held at 175 °C for 5 min and then cooled to 50 °C at a cooling rate of 10 °C/min to establish a standard thermal history. As one of the most important SSA parameters, the self-nucleation temperature (*T*_s_) can be defined as the minimum temperature of melting domains as described by Müller et al. [37]. After the first optimum *T*_s_ of 175 °C was determined by DSC scan at a heating rate of 10 °C/min, the sample was cooled to 80 °C at a cooling rate of 10 °C/min. Afterward, the sample was heated once again to a new *T*_s_ of 170 °C at the same heating rate. The sample was hold at 170 °C for 5 min and then cooled to 80 °C at the same cooling rate. In this step, the fractionation window or temperature interval was determined as 5 °C for the fractionation time during the fractionation process. The above operation was repeated until the entire melting range of the sample was covered. Finally, the sample was heated to 190 °C at the same rate, and the corresponding DSC result could well reflect the influence of total self-nucleation and annealing treatments on the melting behavior of the sample.

The surface morphologies of the fiber samples were identified with a SUPRA 55 field-emission scanning electron microscope (SEM, Carl Zeiss Microscopy GmbH, Jena, Germany). The fiber samples were made electrically conductive by sputter coating with a thin layer of gold-palladium alloy, and the SEM micrographs were taken in a high vacuum mode at an acceleration voltage of 20 kV. The transverse surfaces of the fiber samples were observed by an Olympus BX51 optical microscope (Olympus Corporation, Tokyo, Japan) equipped with a Sony CCD-IRIS digital camera, and some representative digital micrographs were collected. The two-dimensional wide-angle *X*-ray scattering (2D WAXS) and normal *X*-ray diffraction (XRD) patterns of fiber samples were recorded on a Bruker D8 Discover *X*-ray diffractometer (Bruker Nano GmbH, Berlin, Germany) equipped with a GADDS system using Cu *K*_α_ (λ = 0.154 nm) radiation. The tensile properties of fiber samples were measured with an INSTRON 3344 universal testing machine at a tensile speed of 125 mm/min and a grip distance of 250 mm. The thermal degradation behavior of fiber samples was evaluated by thermogravimetric analysis (TGA) on a Pyris–1 thermogravimetric analyzer (Perkin-Elmer Instruments, Richmond, CA, USA) at different heating rates in a nitrogen atmosphere. The hydrolytic degradation behavior was characterized by the retention of mass and tensile strength of the fiber samples immersed in an HCl aqueous solution (10 wt %) and a NaOH aqueous solution (20 wt %) for various hours at room temperature. The thermo-oxidative aging tests were conducted according to the methodology proposed by Singh et al. [38], and the thermo-oxidation coefficient was obtained from the comparison of the tensile properties of fiber samples before and after accelerating thermal aging in an air oven at 70 °C for 72 h.

## 3. Results and Discussion

### 3.1. Melt Spinnability of POM/PLLA Blends

In our previous work, the POM fiber with the tensile strength of 1120 MPa was successfully prepared by a melt-spinning and post-drawing method. This may open a door for the melt-spinning processing of high-strength POM fibers by use of an injection molding-grade copolymerization-type POM resin [18]. Although the potential biodegradation performance and acceptable mechanical properties of POM/PLLA blends have been confirmed by our previous study [35], their spinnability is still unknown. It is well known that the composition of raw materials and spinning-processing conditions are two key factors for melt spinning of POM resin and the associated blends [39,40]. In this work, the melt spinnability of POM/PLLA blends was first evaluated so as to determine the optimal spinning conditions for this blending system. Figure S1 shows the MFRs of POM/PLLA blends at different contents of PLLA. Pure POM is found to have a MFR of 14.9 g/10 min, and however the MFRs of the blends tend to decrease with the incorporation of PLLA. It is noted that the higher PLLA content leads to a lower MFR for the blend. Our previous study verified that the mechanical properties of melt-spun POM fiber were strongly dependent on the MFR of POM resin, and the tensile strength of POM fiber tended to decrease in general due to the minimal loss of spinnability. To minimize the deterioration of spinnability of POM/PLLA blends as well as the mechanical performance of the melt-spun bicomponent fibers, only the blends with the PLLA contents lower than 20 wt % were chosen for further investigation of spinnability.

**Figure 2:**
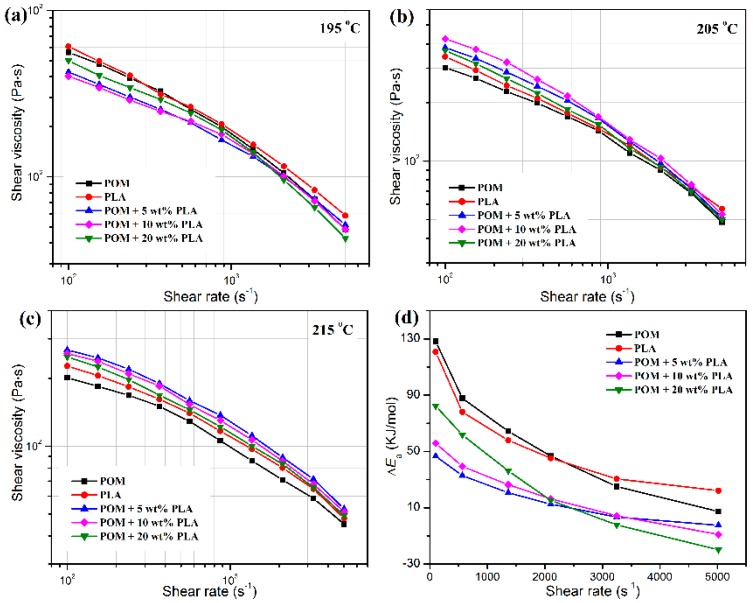
(**a**–**c**) Plots of shear viscosity as a function of shear rate for polyoxymethylene/poly(L-lactic acid) (POM/PLLA) blends at different temperatures; (**d**) flow activation energy of pure POM, PLLA and their blends.

Figure 2 shows the plots of apparent shear viscosities of POM/PLLA blends as a function of the PLLA content at different temperatures, in which both pure polymers and their blends exhibit a pseudo-plastic flow and non-Newtonian behavior [41]. As observed in Figure 2a–c, pure POM and PLLA are found to present similar apparent shear viscosities in the shear-rate range of 100–1000 s^−1^ at 195 °C; however, the apparent shear viscosity of PLLA seems to be higher than that of POM. On the other hand, the POM/PLLA blends exhibit slightly lower apparent shear viscosities in the whole shear rate range compared to two pure polymers, indicating that the presence of PLLA leads to a reduction of shear viscosity due to the slight macromolecular repellency between the two domains caused by partial miscibility. It is noteworthy that the blend containing 20 wt % PLLA present higher apparent shear viscosities in the shear-rate range of 100–2000 s^−1^, but lower viscosities in the higher shear-rate range compared to the other blend samples. These results suggest that the lower PLLA content only facilitates a decrease in apparent shear viscosity at the shear rates lower than 2000 s^−1^ for POM/PLLA blends at 195 °C. With increasing the test temperature, the apparent shear viscosities of pure PLLA are found to be higher than those of pure POM in the whole range of test shear rates as seen in Figure 2b,c. Meanwhile, the POM/PLLA blends show higher apparent shear viscosities than two pure polymers due to the enhanced macromolecular entanglement at higher temperatures. It is noticeable that the blends containing 10 and 5 wt % PLLA present the highest shear viscosities at 205 and 215 °C among the three blend samples, respectively, whereas the blend containing 20 wt % PLLA shows the lowest shear viscosities in the whole range of shear rates.

The activation energy (Δ*E*_a_) of the flow process can be calculated from the slope of ln*η* versus 1/*T* using the Arrhenius equation as expressed by [42]:

(2) $$\eta =A\cdot e^{-\frac{\Delta E_{a}}{RT}}$$

(3) $$\ln\eta =-\frac{\Delta E_{a}}{R}\cdot \frac{1}{T}+\ln A$$

where *η* is the apparent shear viscosity, *A* is a pre-exponential factor, Δ*E*_a_ the activation energy, *T* the absolute temperature (K) and *R* is the universal gas constant (8.314 J·K^−1^·mol^−1^). The obtained values of Δ*E*_a_ are given in Figure 2d. The Δ*E*_a_ of melt is considered as the minimum energy required for macromolecules to just flow, which is equivalent to the energy necessary to overcome the intermolecular forces of attraction as well as the resistance due to the entanglements. Pure POM, PLLA and their blends are all found to show a decrease in Δ*E*_a_ with an increase of shear rate because of the strong shear thinning effectiveness. However, the values of Δ*E*_a_ of the blends are also smaller than any of two pure polymers, indicating that the incorporation of PLLA can effectively enhance the flowability of POM by reducing the energy barrier of molecular motion in the melt state and favors the melt-spinning process accordingly. It is also interestingly found that the Δ*E*_a_ of POM/PLLA blends presents a downward trend by an order of PLLA content of 20 wt % > 10 wt % > 5 wt % in the shear-rate range of 100–2000 s^−1^ but shows an obviously opposite trend in the shear-rate range of 2000–5000 s^−1^. This phenomenon indicates that the higher Δ*E*_a_ is attributed to the greater macromolecular resistance from PLLA domain at higher contents and lower shear rates, and however the disentanglement of PLLA macromolecules may play a major role at higher shear rates, thus resulting in a more significant decrease of Δ*E*_a_ [43]. These rheological results provide an important guideline for designing the optimal melt-spinning processing condition of POM/PLLA blends.

**Figure 3:**
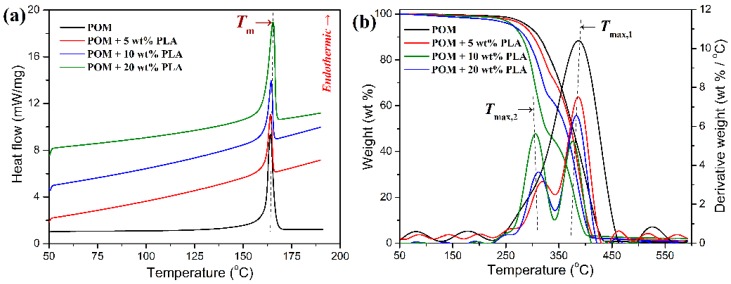
(**a**) Differential scanning calorimetry (DSC), and (**b**) thermogravimetric analysis (TGA) thermograms of POM/PLLA blends at different contents of PLLA.

The melting behavior and thermal stability of POM/PLLA blends were evaluated by DSC and TGA to determine the lower and upper processing temperatures during the melt-spinning process, and the obtained DSC and TGA thermograms are illustrated in Figure 3. It can be observed in Figure 3a that all of the blends showed a single endothermic peak at around 165 °C corresponding to the melting point of POM domain in the blends, and that there is no melting peak found from the PLLA domain in the DSC thermograms of the blends. It is well known that PLLA has a much poorer crystallinity compared to POM, and it is hard to crystallize as a disperse phase in the POM matrix, especially at extremely low loadings due to a confinement effect. In this case, the POM domain can easily perform crystallization in the blends due to its strong crystallization capability, while the PLLA domain is bound to keep in an amorphous state. Therefore, the melt-spinning temperature of POM/PLLA blends is mainly determined by the POM domain. On the other hand, the thermal stability of the blends seems to decrease in the presence of PLLA as observed in Figure 3b. This may be due to the lower thermal decomposition temperature of PLLA domain compared to that of POM one (see the DTG curves in Figure 3b). The perceptible thermal decomposition temperature of POM/PLLA blends is found to locate at about 210 °C. Therefore, the melt-spinning temperature range should be set within 165–210 °C so as to manage adequate fluidity and appropriate thermal stability.

**Figure 4:**
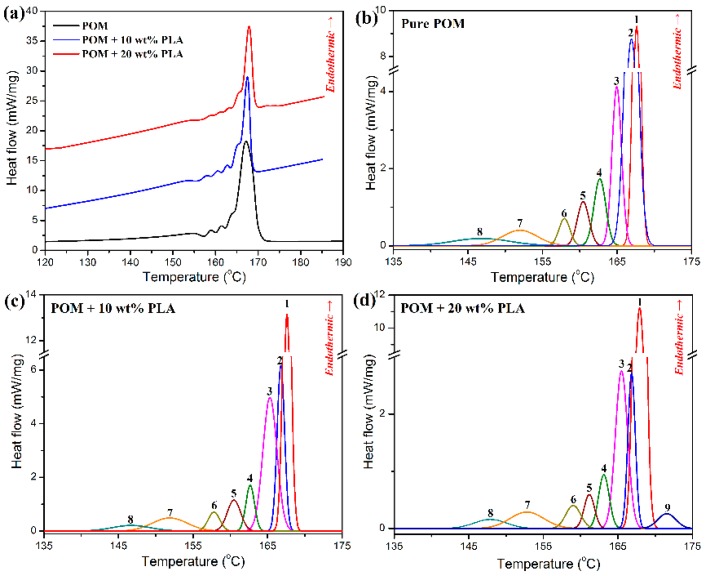
(**a**) DSC survey curves of POM and its blends with PLLA. Successive self-nucleation and annealing (SSA) melting crystallization curves of (**b**) pure POM and its blends with (**c**) 10 wt % and (**d**) 20 wt % of PLLA.

To better determine the post-drawing processing condition for the as-spun fibers derived from POM/PLLA blends, the SSA thermal fractionation experiments were conducted to understand the crystallization characteristics of the blends [44]. Figure 4a shows the survey DSC curves obtained from thermal fractionation experiments for pure POM and its blends with PLLA, and the representative SSA thermal fractionation curves are illustrated in Figure 4b–d. As observed in Figure 4a, the survey DSC curve of pure POM presents multiple melting behaviors corresponding to the number of SSA cycles, and each melting endothermic peak represents the melting of crystallites formed from the POM macromolecules with the similar oxymethylene sequence length and lamellar thickness [45], indicating that there is heterogeneity existing in POM resin [46]. Although the POM/PLLA blends exhibit the similar survey fractionation DSC curves with pure POM, it can also be identified that the SSA peaks in the blends mainly arise from the fraction crystallization of POM domain by comparing Figure 4b with Figure 4c,d. Considering the fact that each SSA peak is proportional to the weight fraction of the crystallites with the same stability, the relative contents of these crystallites can be derived from the differential normalized area under the fusion of pure POM and its blends with PLLA. The detailed data obtained from the SSA thermal fractionation analysis are summarized in Table S1 (see Supplementary Materials). It is noted that pure POM exhibits eight well-resolved melting peaks resulting from the crystallization at 155–170 °C, and three major fractions at 167.6, 166.9, and 165.0 °C contribute more than 78% of the crystallites in the thermal fractionation. Similarly to the SSA thermal fractionation result of pure POM, the POM/PLLA blends also present three major fractions at similar temperatures contributed by the crystallization of POM domain. However, the relative content of the fraction at 167 °C is improved with an increase of PLLA content, and the relative contents of the fractions at the other two temperatures tend to decrease. This may be ascribed to the disturbance of crystallization of POM phase in the presence of PLLA domain. Such a disturbance becomes more and more serious with an increase of PLLA content and finally leads to a new small fraction of crystallites appearing at 171.6 °C for the blends containing 20 wt % PLLA. Based on the results of SSA thermal fractionation experiments for POM/PLLA blends, a gradual slow cooling program with a temperature range from 170 °C down to 155 °C were determined to offset the crystallization of POM domain in the blends.

### 3.2. Morphology and Microstructure

**Figure 5:**
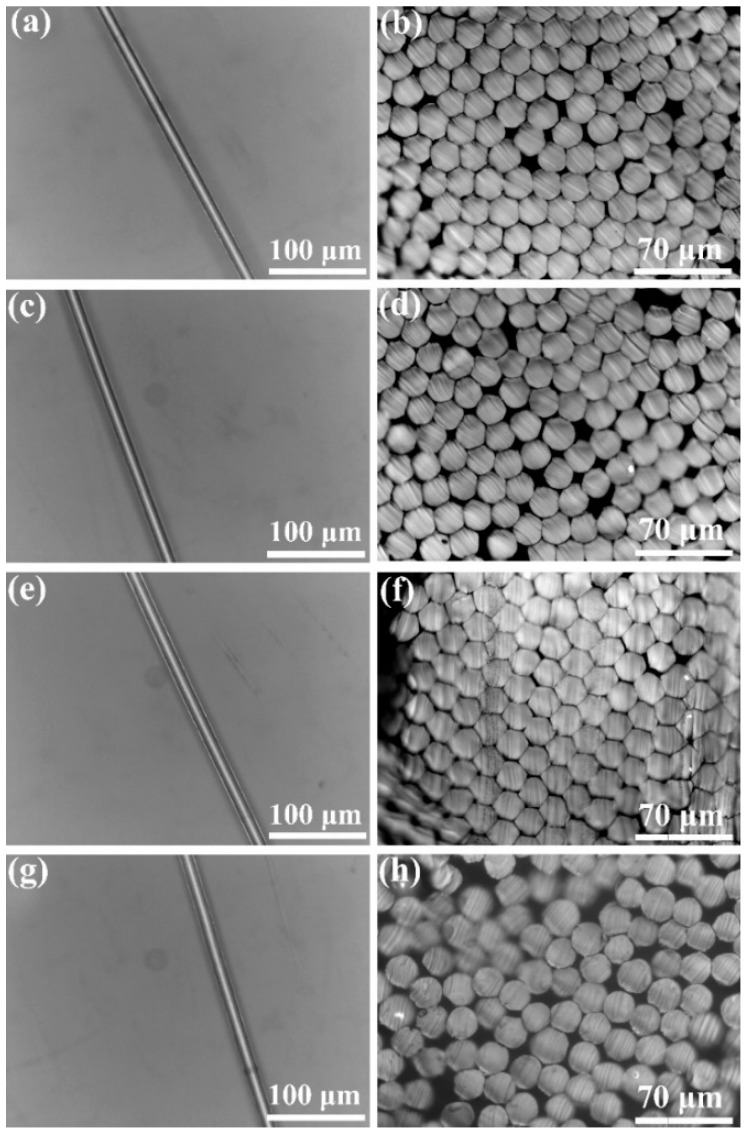
Optical microscopic images of filament (left) and transverse surfaces (right) of (**a**,**b**) pure POM fiber and POM/PLLA bicomponent fibers containing (**c**,**d**) 5, (**e**,**f**) 10, and (**g**,**h**) 20 wt % PLLA at the ultimate draw ratio.

Figure 5 shows the optical microscopic images of single filament and transverse surfaces of the melt-spun fiber samples obtained at the ultimate draw ratios. Both pure POM fiber and POM/PLLA bicomponent fibers are found to exhibit a homogenous profile and a uniform diameter distribution. Moreover, there is also no sign of filament spitting observed from the bicomponent fibers. It is known that the POM resin used in this work has a good balance between the crystalline and amorphous phases to facilitate the melt spinning and subsequent post-spinning processing, thus leading to the formation of perfect filaments. The aforementioned melting behavior revealed that only the POM domain performed crystallization in the POM/PLLA blends, and the PLLA domain is partially miscible with the POM one in the amorphous phase. This might enhance the interfacial adhesion of the POM and PLLA phases and therefore made the as-spun bicomponent fibers undergo an ultimate tensile stress like as-spun pure POM fiber under the post-drawing process [47]. In addition, the optical microscopic observation is also indicative of the fact that the incorporation of PLLA seems not to influence the diameter distribution and structure of filaments.

**Figure 6:**
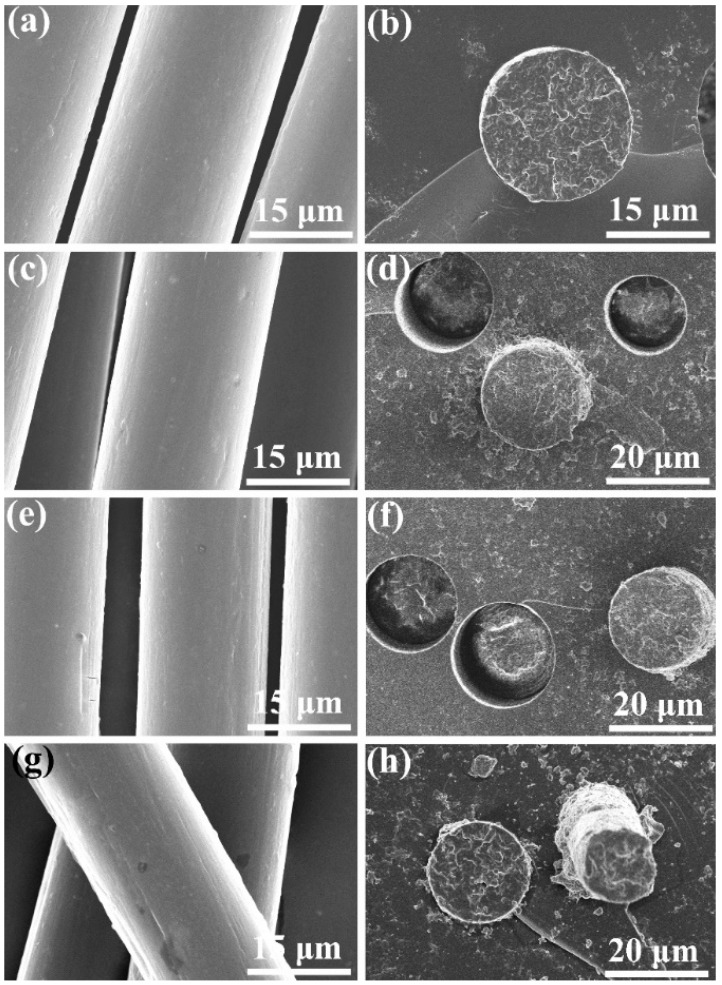
SEM micrographs of profiles (left) and transverse surfaces (right) of (**a**,**b**) pure POM fiber and POM/PLLA bicomponent fibers containing (**c**,**d**) 5, (**e**,**f**) 10, and (**g**,**h**) 20 wt % PLLA at the ultimate draw ratios.

**Figure 7:**
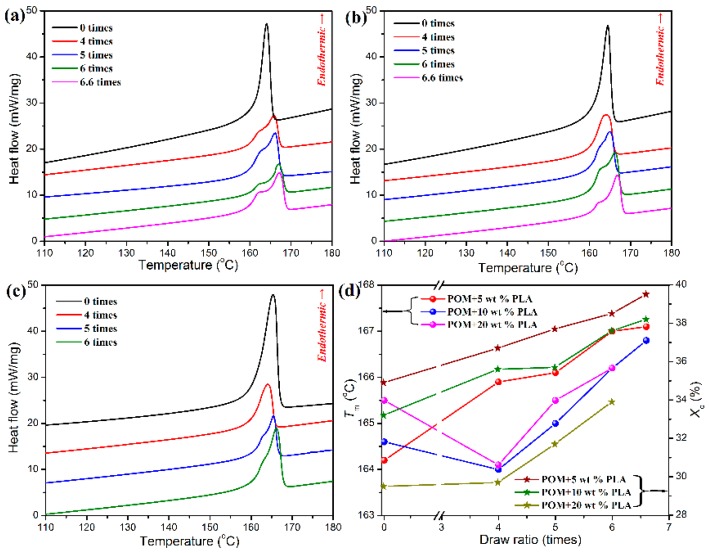
DSC thermograms of POM/PLLA bicomponent fibers containing (**a**) 5, (**b**) 10, and (**c**) 20 wt % PLLA at different draw ratios; (**d**) melting temperatures and degree of crystallinity of POM/PLLA bicomponent fibers at different draw rations.

To further identify the morphology and microstructure of the bicomponent fibers, the profiles and transverse surfaces of fiber samples were observed by SEM, and the obtained micrographs are displayed in Figure 6. As seen in Figure 6a, POM fiber exhibits a smooth and clear surface almost with no collapse or grooves on the filament surface. Meanwhile, a compact texture can be observed on the transverse surfaces, and there are no microcracks and microvoids found, as seen in Figure 7b, indicating the successful formation of POM fiber under the current melt-spinning and post-drawing technologies. The filament diameter of POM fiber is determined as approximately 18 μm according to the transverse morphology in Figure 6b. On the other hand, the bicomponent fibers are also found to display a smooth surface morphology with few of defects; however, a small quantity of speckles can be noted on the surface of the bicomponent fibers obtained from the blends containing 20 wt % PLLA due to the coagulation of the PLLA phase on the filament skin caused by exclusion of POM crystallites during the annealing process of as-spun fibers [48]. Nevertheless, the bicomponent fibers still present a compact and homogenous texture as observed from the SEM micrographs of transverse surfaces in Figure 6d,f, and h, and there is no distinguishable defect or phase separation found on their transverse surfaces. Moreover, the filament diameters of three bicomponent fiber samples are found to be similar with each other, and can be determined as 18–20 μm. In summary, the morphological observations confirm that the bicomponent fibers have been successfully fabricated by melt spinning and post drawing due to the partial miscibility between POM and PLLA.

### 3.3. Crystallization Characteristics and Orientation Structure

Although the bicomponent fibers could be well melt spun by using the POM/PLLA blends containing 5, 10, and 20 wt % PLLA, the three fiber samples are found to undertake different ultimate draw ratios during the post-drawing process. Actually, the bicomponent fibers containing 5 and 10 wt % PLLA can undergo an ultimate draw ratio of 6.6 times, whereas the bicomponent fiber containing 20 % PLLA can merely be drawn ultimately by 6.0 times. This phenomenon may be associated with the crystallization characteristics of bicomponent fibers. The crystallization histories of the bicomponent fibers at different draw ratios can be reflected by their melt behaviors recorded in the DSC heating thermograms as shown in Figure 7a–c. It is important to note in these DSC thermograms that the post-drawn bicomponent fibers exhibit different melting behaviors from the as-spun ones. All of the as-spun bicomponent fibers only show a single endothermic peak in their DSC thermograms. As confirmed by the aforementioned melting behavior of POM/PLLA blends, only the POM matrix is able to perform crystallization due to the poor crystallinity of PLLA disperse phase in the matrix. Such a single endothermic peak is attributed to the melting process of the POM domain in the bicomponent fibers. It is well known that a large number of folded-chain crystallites as well as a few of extended-chain crystallites are bound to form during the cooling process of as-spun semi-crystalline polymeric fibers [7], whereas the occurrence of single melting peak implies a similar crystal size for the folded-chain crystallites of POM domain in the as-spun bicomponent fibers. Although the bicomponent fibers containing 5 and 10 wt % PLLA show a single melting peak in the DSC thermograms after post-drawn at a draw ratio of four times, a bimodal melting behavior is observed for other post-drawn bicomponent fibers at any draw ratios. Such a bimodal melting behavior is ascribed to the presence of the doublet crystal state of POM domain. Considering the more stable structure of extended-chain crystallites than the folded-chain ones, it is believable that the low-temperature endothermic peak is related to the folded-chain crystallites with a smaller lamellar thickness as well as to the imperfect crystallites, and the high-temperature one is associated with the extended-chain ones [49]. The bimodal DSC thermograms were further numerically deconvolved to determine the real contribution of both endothermic components, and the resulting deconvolved curves are presented in Figure S2 (see Supplementary Materials). It is noticeable that, in the most cases, the contribution from the low-temperature endothermic component is improved with an increase of draw ratio, indicating an increase of the quantity of imperfect crystallites and a decrease of the lamellar thickness of folded-chain crystallites caused by the increased draw ratio.

**Figure 8:**
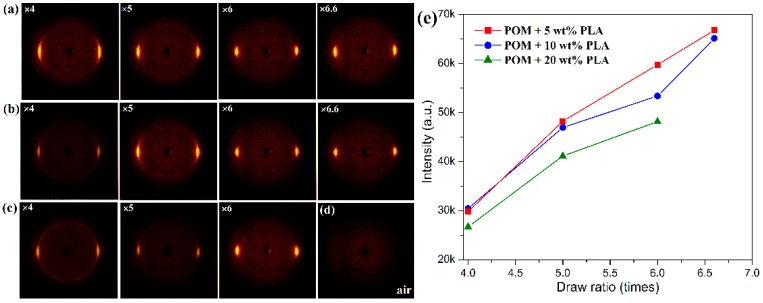
Two-dimensional wide-angle X-ray scattering (2D WAXS) patterns of POM/PLLA bicomponent fibers containing (**a**) 5, (**b**) 10, and (**c**) 20 wt % PLLA at different draw ratios, and (**d**) air as a reference; (**e**) plots of the azimuthal diffraction intensity at 2θ = 23.05° on the equator plane for the (100) reflection of the bicomponent fibers.

The melting temperatures (*T*_m_’s) of bicomponent fibers obtained from DSC analysis are given in Figure 7d. The post-drawn bicomponent fibers containing 5 wt % PLLA are found to show a continual improvement in melting temperature (*T*_m_) with an increase of draw ratio, indicating that a large number of folded-chain crystallites have transformed into the extended-chain crystallites during the post-drawing process for the as-spun bicomponent fibers [8]. Nevertheless, there is a significant decline in *T*_m_ for the bicomponent fibers containing 10 and 20 wt % PLLA at a draw ratio of four times, and then the *T*_m_’s of the two fiber samples tend to increase with the increasing content of PLLA. This phenomenon may be due to the production of imperfect crystallites resulting from high PLLA loadings in the bicomponent fibers during the initiate drawing stage, followed by the presence of a quantity of extended-chain crystallites under a further hot-drawing condition. Figure 8d shows the degrees of crystallinity of POM phase in the bicomponent fibers at different draw ratios. It is notable that all of the bicomponent fibers exhibit an upward trend in degrees of crystallinity with an increasing of draw ratio, which is attributed to the stress-induced crystallization. However, such a stress-induced crystallization effect seems to be not so significant for the bicomponent fibers containing 20 wt % PLLA at the draw ratio of four times because of the disturbance of PLLA phase. Only a higher draw ratio can compensate the stress-induced crystallization on the POM domain, thus leading to an improvement in degree of crystallinity for the bicomponent fibers at a higher content of PLLA.

**Figure 9:**
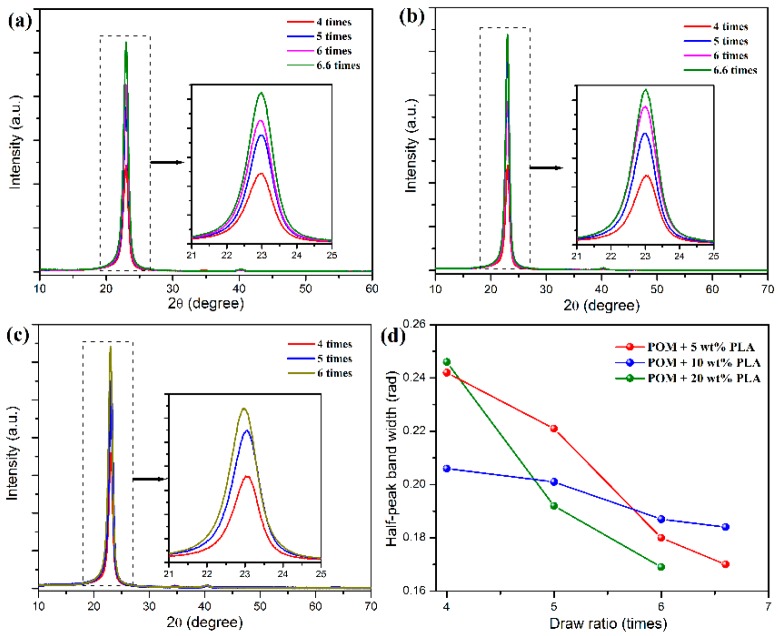
Azimuthal intensity distributions on the equator plane of XRD patterns of POM/PLLA bicomponent fibers containing (**a**) 5, (**b**) 10, and (**c**) 20 wt % PLLA at different draw ratios; (**d**) plots of half-peak band width as a function of draw ratio for POM/PLLA bicomponent fibers.

The characteristic crystallization structures of POM/PLLA bicomponent fibers at different draw ratios were identified by 2D X-ray diffraction, and the 2D WAXS patterns and the recorded azimuthal intensity distributions on the equator plane of registered images are illustrated in Figure 8 and Figure 9, respectively. It is clearly observed in Figure 8a–c that the shape of the scattering in the 2D WAXS patterns varies significantly with an increase of draw ratio. The 2D WAXS patterns exhibit a combination of a strong maximum on the equator and a weak scattering ring, indicating the occurrence of orientation of POM crystals along the draw direction. Furthermore, the strong maximum is ascribed to the diffraction of the particular Bragg angle of 23.05° for the (100) reflection. The brightness of the scattering ring is found to decrease and disappear at the ultimate draw ratio due to the reduction of diffraction intensity at the other reflection. Finally, only two bright arcs or spots intersecting the equator and extending systematically above and below the equator are observed in the 2D WAXS patterns. It is also noted in Figure 9a–c that an intensive diffraction peak could be observed at 2θ = 23.05° from the azimuthal intensity distributions on the equator plane, which is assigned to the (100) reflection of the crystals of the POM domain. The diffraction intensity of the (100) plane is directly associated with the uniaxial crystalline orientation level of the POM crystals. It is discernible from the inserts of this figure that the diffraction intensity on the (100) reflection tends to be enhanced continuously by improving the draw ratio for all of the bicomponent fibers. The diffraction peak width of three fiber samples is also found to become smaller with an increase of the draw ratio due to the improvement of the continuity level in the alignment of crystallites along the draw direction, as observed in Figure 9d [50]. Moreover, Figure 8e shows the diffraction intensity at the (100) reflection for the bicomponent fibers at the different draw ratios. It is observed that the diffraction intensity at the (100) reflection tends to increase with an increase of the draw ratio, indicating an enhancement of orientation level along the draw direction. These results indicate that the lamellar crystallites of POM domain are preferentially oriented perpendicular to the draw direction with a small portion of isotropic macromolecules. Furthermore, the brightening arcs or spots implicates that the bicomponent fibers gain a high degree of orientation due to the strain-induced reorganization of lamellar crystallites in the POM domain caused by uniaxial drawing [51]. Such a high orientation level may facilitate to endow the POM/PLLA bicomponent fibers with a high elastic modulus and high tensile strength.

**Figure 10:**
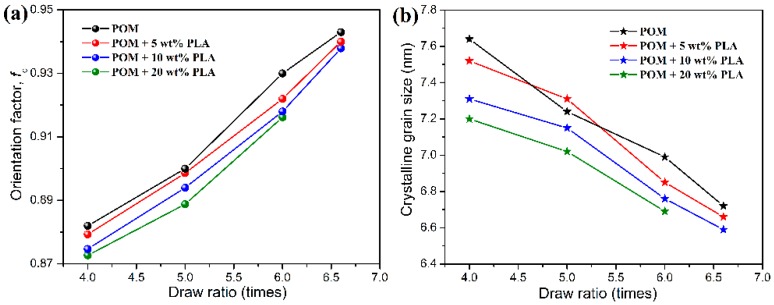
(**a**) *C*-axis crystalline orientation factors, and (**b**) grain sizes of pure POM fiber and POM/PLLA bicomponent fibers at different draw ratios.

According to the crystallographic study, the POM crystal normally has a hexagonal structure with the unit cell dimensions of *a* = *b* = 4.45 Å and *c* = 17.3 Å, their molecular chains are arranged in a 9/5 helix, where the *a*– and *b*–axes of the crystal are located on the same plane and its *c*–axis is perpendicular to that plane [52]. Some important information about crystalline orientation can be provided by the 2D XRD characterization for the drawn bicomponent fibers. It was reported that the crystalline orientation factor (*f*_c_) along the draw direction could be determined quantitatively by the Hermans orientation model as generalized to a set of three crystallographic axes, and it could be expressed by the following equation [53]:

(4) $$f_{c}=\frac{3\langle \cos^{2}\phi \rangle -1}{2}$$

where <cos^2^*ϕ*> is the mean-squared cosine of the angle between the reference crystallographic axis and the selected reference direction (fiber *c*–axis) for a fiber sample. For a uniaxially oriented polymeric fiber, the value of <cos^2^*ϕ*> can be calculated by the following equation [7,50]:

(5) $$\langle \cos^{2}\phi \rangle =\frac{\int_{0}^{\frac{\pi }{2}}I(\phi )\cos^{2}\phi \sin\phi d\phi }{\int_{0}^{\frac{\pi }{2}}I(\phi )\sin\phi d\phi }$$

where *I*(*ϕ*) is the relative intensity scattered from the (*hkl*) reflections which are normalized to the *c*–axis through the Gaussian fitting. As for the drawn bicomponent fibers, the (100) reflection corresponding to the *c*–axis can be used to calculate the *c*–axis crystalline orientation factor (*f*_c_) [54]. Considering the hexagonal structure and uniaxial symmetry of the unit cell, the orientation factors of the *a*- and the *b*-crystallographic axes are identical to each other (*f*_a_ = *f*_b_). Since *f*_a_ + *f*_b_ + *f*_c_ = 2*f*_a_ + *f*_c_ = 0, the uniaxial crystalline orientation factors of the bicomponent fibers can be determined by an azimuthal scan of the (100) reflection at 2θ = 23.05°. Figure 10a shows the uniaxial crystalline orientation factors of pure POM fiber and POM/PLLA bicomponent fibers at different draw ratios. As seen in Figure 10a, both pure POM fiber and the bicomponent fibers are found to exhibit a continual increase of crystalline orientation factor with the increasing draw ratio, indicating that the crystalline orientation of lamellar folded-chain crystallites in the POM phase occurs under uniaxial stretching. The higher draw ratio can promote a greater level of crystalline orientation and therefore leads to a continual increase in crystalline orientation factor for pure POM fiber and the bicomponent fibers. It is interestingly observed that the bicomponent fibers have smaller orientation factors compared to pure POM fiber at the same draw ratio, and however the bicomponent fibers with a lower content of PLLA present a greater orientation factor. This result suggests that the higher PLLA content not only can suppress the crystalline packing of the POM phase, but also that it can hinder the stress-induced orientation of the lamellar folded-chain crystallites, thus restraining the increase of crystalline orientation level for the bicomponent fibers.

Besides the crystalline orientation factor, the crystalline grain size can also be calculated by the Scherrer’s equation according to the Bragg diffraction angle and full width at half maximum of diffraction peak obtained from WAXD patterns. The Scherrer’s equation is well known as a primary model to determine the lamellar thickness in polymeric crystallites and can be expressed as [55,56]:

(6) $$L_{hkl}=\frac{K\lambda }{\beta \cos\theta }$$

where *L*_hkl_ represents the mean size of the ordered crystalline domains almost equal to the crystalline grain size, *K* the dimensionless shape factor generally taken as 0.89, *λ* the applied *X*-ray wavelength, *θ* the Bragg angle, and *β* is the full width at half maximum diffraction intensity. Figure 10b shows the crystalline grain sizes corresponding to the (100) reflection of POM crystals in pure POM fiber and POM/PLLA bicomponent fibers at different draw ratios. As observed in Figure 10b, the crystalline grain size is found to decrease gradually with an increase of draw ratio, indicating that the post-drawing process leads to the rupture of crystallites and then the slippage of the lamellar folded-chain crystallites in the crystallites [56]. It is understandable that a higher draw ratio can promote the more significant slippage of crystallites, thus leading to a smaller crystalline grain size and higher orientation level. In addition, it is also found that the bicomponent fibers at a higher PLLA loading exhibit a smaller crystalline grain size at the same draw ratio. This can be explained by the fact that the high PLLA loading can depress the growth of crystallites in the bicomponent fibers during the post-drawing process, thus reducing the crystalline grain size accordingly.

### 3.4. Mechanical Properties

**Figure 11:**
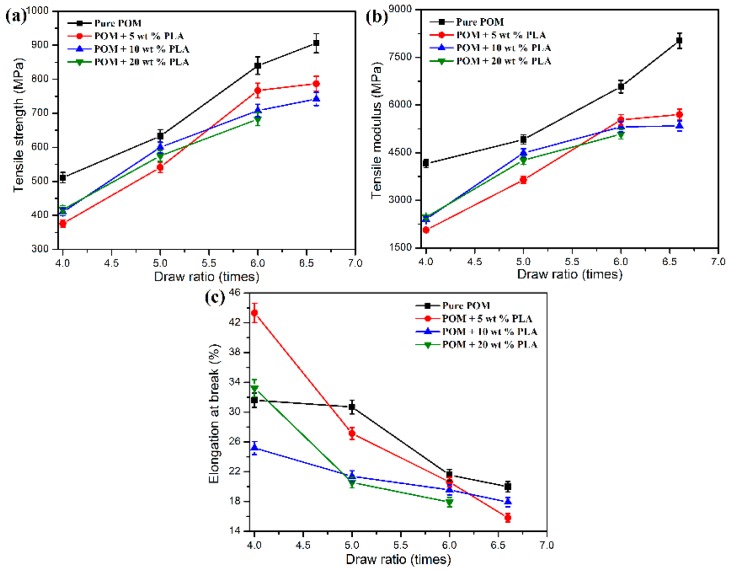
(**a**) Tensile strength, (**b**) tensile moduli, and (**c**) elongation at break of pure POM fiber and POM/PLLA bicomponent fibers at different draw ratios.

The effects of the PLLA content and draw ratio on the tensile properties of POM/PLLA bicomponent fibers are demonstrated in Figure 11, in which the mechanical data of pure POM fiber are also presented as a reference. As seen in Figure 11, the draw-ratio dependency of the tensile performance is observed from both pure POM fiber and POM/PLLA bicomponent fibers. For all of the fiber samples, both the tensile strength and elastic modulus are found to increase almost linearly with the draw ratio. The improvement in tensile strength and elastic modulus is attributed to the crystalline orientation as well as the molecular orientation in the amorphous region during the stretching process. The post drawing leads to a transformation from the folded-chain crystallites into the extended-chain ones, and meanwhile the stress-induced crystallization promotes an improvement of the degree of crystallinity. In this case, the extended-chain crystallites make a major contribution to the enhanced tensile properties along the draw direction. It is noteworthy that the linear relationship between the draw ratio and tensile performance becomes poorer for the bicomponent fibers, which may be ascribed to the obstruction of crystalline orientation in the presence of PLLA phase in the bicomponent fibers. The crystalline orientation is almost completed at the ultimate draw ratio. Pure POM fiber achieved a high elastic modulus of 8024 MPa and high tensile strength of 903 MPa at the ultimate draw ratio of 6.6 times. However, the presence of the PLLA domain results in a decrease of tensile performance due to the reduction of crystalline orientation caused by the alien obstruction for the drawn fibers. It is understandable that the higher the content of PLLA, the more significant the alien obstruction effect, and therefore a lower elastic modulus and tensile strength are achieved for the bicomponent fibers. Owing to such an alien obstruction effect, only an ultimate draw ratio of six times is available for the bicomponent fibers containing 20 wt % PLLA. Nevertheless, the bicomponent fibers still obtained the tensile strength of 787 and 742 MPa at the PLLA contents of 5 and 10 wt %, respectively. Such the mechanical data are still accepted for the applications of synthetic fibers. In addition, it is noted in Figure 11c that the elongation at break exhibits a downward trend with an increase of draw ratio for both pure POM fiber and POM/PLLA bicomponent fibers. This phenomenon is due to the fact that the high crystalline orientation can promote the rupture and slippage of lamellar crystallites and therefore leads to the formation of microvoids during the solid-phase deformation, thus resulting in a failure in the further uniaxial deformation of filaments. Anyway, the drawn bicomponent fibers that can undertake the ultimate draw ratio of 6.6 times still present enough mechanical performance for further applications.

### 3.5. Hydrolytic Degradation Behavior

**Figure 12:**
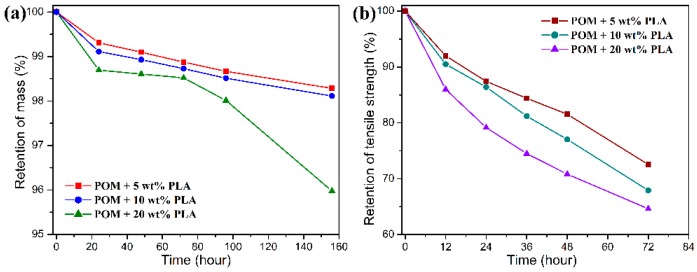
(**a**) Mass, and (**b**) tensile strength retention as a function of degradation time for POM/PLLA bicomponent fibers in an acid medium.

**Figure 13:**
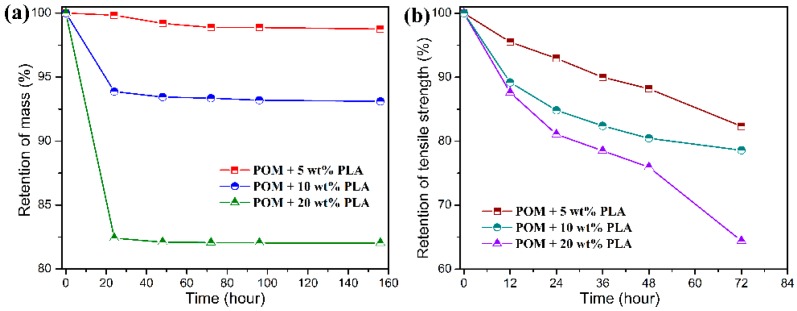
(**a**) Mass, and (**b**) tensile strength retention as a function of degradation time for POM/PLLA bicomponent fibers in an alkali medium.

The hydrolytic degradation measurement was conducted as an accelerated simulation experiment to evaluate the biodegradability of POM/PLLA bicomponent fibers. It has been reported that the degradation of PLLA is primarily due to the hydrolysis of the ester linkages, which occurs more or less randomly along its macromolecular backbone according to the following diffusion-reaction mechanism [57]: the water penetrates the PLLA matrix and simultaneously converts the long molecular chain to low-molecular-weight water-soluble oligomers and finally the given monomers. The resultant degradation products have an increased density of polar groups (hydroxyl and carboxyl) compared to the initial polymer and induce an enhanced water affinity promoting the degradation reaction. Meanwhile, the ester bond hydrolysis can be auto-catalyzed by any carboxylic end groups initially formed during the hydrolysis process. As for the POM/PLLA bicomponent fibers, the hydrolysis of PLLA domain can produce the microvoids within the filaments, thus leading to the collapse and pulverization of filaments. As a result, the partial biodegradability is achieved for the bicomponent fibers. Figure 12 and Figure 13 show the mass and tensile strength retention of POM/PLLA bicomponent fibers after being soaked in the acid and alkali media for certain periods, respectively. It is important to note in Figure 12 that all of the bicomponent fibers show a great loss in tensile strength after soaked in the acid medium for 72 h, whereas a high mass retention over 98% is maintained for these fiber samples. With the extension of soaking time, the mass retention tends to decrease rapidly. This suggests that the initiate hydrolysis of PLLA domain only damages the structure of filaments and therefore deteriorates the tensile performance, and the follow-up hydrolytic degradation reaction leads to the mass loss of the bicomponent fibers. It seems that the retention of mass and tensile strength is lower for the bicomponent fibers with a higher content of PLLA over the same degradation period, indicating that there are more PLLA molecules involved in the hydrolysis under the acidic condition.

Although there is a significant decrease in the retention of tensile strength for the bicomponent fibers under the alkali medium, the degradation rate is found to be much faster than that in the acidic medium, resulting in a complete hydrolytic degradation of PLLA domain within 20 h as observed in Figure 13. In this case, the mass retention presents a drastic decrease in accordance to the PLLA loading in the bicomponent fibers within 20 h and then tends to flat with the further extension of sacking time. Such a hydrolytic degradation disintegrates the filaments and thus results in the decrease of tensile strength. It is noteworthy that the bicomponent fibers seen to show a rapider degradation rate in the alkali media than in the acid one. This can be explained by the fact that the degradation rate of PLLA is more easily accelerated by the formation of dissociated form of lactic acid as well as the splitting of lactoyl lactate in the alkali media [58]. Therefore, the ester characteristics of PLLA domain impact a hydrolytic degradability to the POM/PLLA bicomponent fibers, and the adjustment of hydrolytic degradation behavior can also be expected for the bicomponent fibers on the basis of the PLLA loading and pH value of medium.

### 3.6. Thermo-oxidative Aging Performance

**Figure 14:**
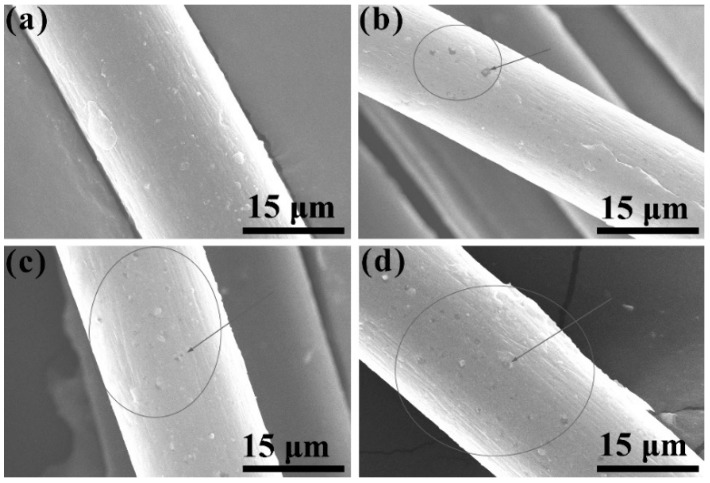
SEM micrographs of the surfaces of (**a**) pure POM fiber and POM/PLLA bicomponent fibers containing (**b**) 5, (**c**) 10, and (**d**) 20 wt % PLLA at the ultimate draw ratio after thermo-oxidation aging.

Although the blending of POM and PLLA can well establish a type of partially biodegradable material for specific end uses, the biodegradable POM/PLLA bicomponent fibers exposed to heat may be subject to a series of physical and chemical changes. The short exposure times at elevated temperatures generally serve to shorten the induction period of the oxidatively degradable synthetic fibers. The physical properties including tensile strength and elongation at break may change during this induction period. Nevertheless, such the changes are usually not only caused by structural changes of molecular chains but also are due to a temperature-dependent response. In this case, the loss of tensile properties is ascribed to the embrittlement of the bicomponent fibers and can be evaluated at the different temperatures corresponding to the relevant time scales [37]. The thermos-oxidative aging behavior of the bicomponent fibers can be characterized by means of the so-called thermo-oxidation coefficient (*K*_TO_), which is calculated according to the tensile properties before and after the thermo-oxidation aging and expressed as:

(7) $$K_{TO}=\frac{v_{a}}{v_{0}}$$

where *v*_0_ and *v*_a_ are the values of tensile strength or elongation at break before and after the thermo-oxidation aging test, respectively. Table S2 gives the data of *K*_TO_ according to the tensile strength and elongation at break for pure POM fiber and POM/PLLA bicomponent fibers at different draw ratios after an accelerated aging process in a hot air at 70°C for 72h. As seen in Table S2, pure POM fiber keeps 98% of its tensile strength and 97% of elongation at the break at the ultimate draw ratio, indicating an excellent thermo-oxidative aging property due to the high degree of crystallinity of POM. However, there is a slight decrease in the data of *K*_TO_ according to the tensile strength and elongation at break with an increase of draw ratio. This may be due to the presence of amorphous regions on the surface of POM fiber after hot drawing. It is understandable that the amorphous POM can be thermally oxidated more easily than the crystalline one, because the atmospheric oxygen is easily diffused into the inside of fibers through the amorphous region of molecular chains [59]. The higher draw ratio leads to more amorphous regions appearing on the fiber surface and results in more defects after thermo-oxidative aging accordingly. As a result, the tensile performance tends to deteriorate with the improvement of draw ratio. The bicomponent fibers are found to exhibit a relatively poorer thermo-oxidative aging property compared to pure POM fiber as observed in Table S2, and the data of *K*_TO_ also tend to decrease with an increase of PLLA content. It is evident that the amorphous regions on the fiber surface fairly increase in the presence of amorphous PLLA phase. On the other hand, the amorphous regions can be further increased as the content of PLLA increases. These increased amorphous regions are obviously more disadvantageous to the thermo-oxidative aging property of the bicomponent fibers. Figure 14 displays the SEM micrographs of the surfaces of pure POM fiber and POM/PLLA bicomponent fibers after the thermo-oxidative aging experiment. It is noticeable that there are more defects distinguished on the surface of the bicomponent fibers after the thermo-oxidative aging compared to pure POM fiber. The number of defects seems to increase with an increase of PLLA content as observed in Figure 14b–d. The amorphous PLLA phase is more prone to deteriorate than the POM one when exposed to the thermo-oxidative environment, resulting in more defects on the fiber surface accordingly. The presence of these defects leads to the worse tensile performance of the bicomponent fibers. Nevertheless, the bicomponent fibers still maintain almost 90% of tensile strength after the thermo-oxidative aging, promising fairly good thermo-oxidative aging performance.

### 3.7. Thermal Degradation Behavior and Kinetics

**Figure 15:**
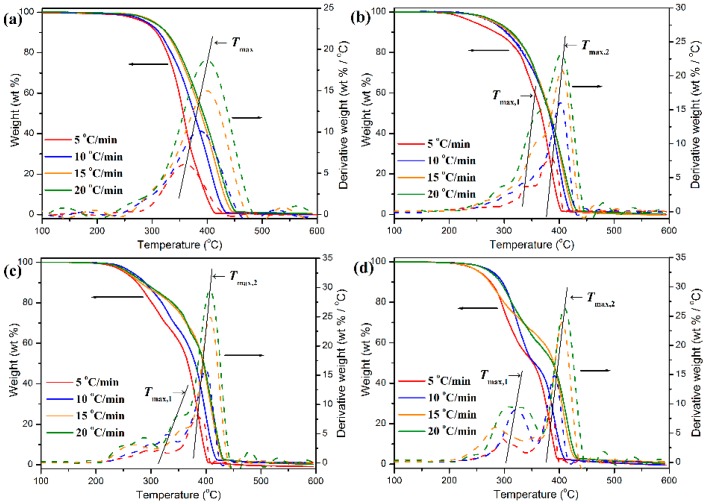
TGA and DTG thermograms of (**a**) pure POM fiber and POM/PLLA bicomponent fibers containing (**b**) 5, (**c**) 10, and (**d**) 20 wt % PLLA at the ultimate draw ratios.

The thermal degradation behaviors of pure POM fiber and POM/PLLA bicomponent fibers were studied by TGA at different heating rates, and the obtained TGA and DTG thermograms are illustrated in Figure 15. To achieve a better insight into the thermal decomposition process, some important decomposition parameters derived from the TGA characterizations were summarized in Table S3 (see Supplementary Materials), which include the initial degradation temperature (*T_i_*) corresponding to 5 wt % weight loss, the final degradation temperature (*T_f_*) corresponding to 5 wt % residual char left, and the characteristic temperature (*T*_max_) at the maximum weight-loss rate. As seen in Figure 15, pure POM fiber presents a typical one-stage decomposition behavior in the temperature ranges of 285–450 °C at the given heating rates due to the pyrolysis of main chains of POM. It is noteworthy in Table S3 that there is a shift to higher temperatures occurring in *T_i_*, *T_f_*, and *T*_max_ with an improvement of heating rate, indicating that pure POM fiber has to complete thermal degradation in a higher temperature region due to the slow heat diffusion in the faster heating process [60]. On the contrary, the POM fiber must achieve an equilibrium in thermal degradation rapidly during the slower heating process at a low heating rate, and therefore it completes the thermal decomposition in a lower temperature region. However, a two-step thermal degradation behavior is observed from the TGA thermograms of the bicomponent fibers, in which the earlier weight loss corresponds to the decomposition of the PLLA domain and the later one is attributed to the degradation of the POM domain. The earlier weight loss is dependent on the PLLA loading in the bicomponent fibers, and the higher PLLA content actually leads to a greater weight loss at the earlier decomposition stage. Moreover, there are two *T*_max_’s observed in the DTG thermograms of the bicomponent fibers, in which the lower peak temperature (*T*_max,1_) is attributed to the PLLA domain, and the higher peak (*T*_max,2_) is associated with the thermal degradation of the POM domain. It is well known that the thermal stability of a polymer is directly related to its crystallinity. The TGA results well identified that the crystalline POM phase had a much better thermal stability than the amorphous PLLA one in the bicomponent fibers. In is important to note that the *T*_max,2_ seems to increase fairly with the content of PLLA at the same heating rate, which may be ascribed to the enhancement in both the crystallinity of POM domain and the envelopment effect of PLLA domain. It is understandable the high crystallinity can improve the heat resistance of the POM domain. On the other hand, the envelopment with PLLA domain can effectively retard the heat diffusion into the POM domain. Such a dual effect enhances the thermal stability of the POM domain in the bicomponent fibers. However, the *T*_max,1_ is found to decrease slightly with an increase of PLLA content at the same heating rate, which may be due to the increased loading of the thermally unstable PLLA domain in the bicomponent fiber. In addition, it is noteworthy in Figure 15 and Table S3 that with a decrease of heating rate, a downward trend is observed in both *T*_max,1_ and *T*_max,2_ for all of the bicomponent fiber samples, suggesting a poorer thermal stability for these bicomponent fiber samples at a lower heating rate due to the longer heating period.

The decomposition kinetics of the pure POM fiber and POM/PLLA fibers was further studied by means of the Kissinger’s and Flynn-Wall’s methods in this work. According to the decomposition reaction of a polymer during the thermal degradation process, the conversion rate (*a*) of decomposition reaction is defined as the ratio of mass loss at arbitrary time to total mass loss at the complete decomposition temperature from TGA analysis, and it is calculated by the following equation:

(8) $$\alpha =\frac{w_{0}-w_{t}}{w_{0}-w_{f}}$$

where *w*_0_, *w*_t_ and *w*_f_ are the initial weight, actual weight at time *t*, and final weight of the sample at the end of thermal decomposition, respectively. The decomposition kinetic equation can further be rewritten by Equation (10) in terms of the reaction rate for a basic solid-state chemical reaction [61]:

(9) $$d\alpha /dT=k{\left(1-\alpha \right)}^{n}$$

where *T* is the reaction temperature, *k* the rate constant of thermal decomposition, and *n* represents the apparent order of reaction. Moreover, the relationship between the rate constant and the apparent activation energy (Δ*E*_a_) can be established by the Arrhenius’s equation:

(10) $$k=A\exp(-\Delta E_{a}/RT)$$

The combination of Equations (9) and (10) with the Arrhenius expression gives the following relationship:

(11) $$d\alpha /dT=A{\left(1-\alpha \right)}^{n}\exp(-\Delta E_{a}/RT)$$

This relationship is determined as the basis of numerous analytical approaches to the calculation of kinetic parameters from TGA results. Table S4 summarizes the thermal decomposition temperatures of the pure POM fiber and POM/PLLA bicomponent fibers at different conversion rates (see Supplementary Materials). It is noted in Table S4 that the introduction of PLLA seems to promote the reaction evolution of the bicomponent fibers in the earlier decomposition stage due to the poorer thermal stability of PLLA domain compared to the POM domain. Meanwhile, the degradation temperatures of the bicomponent fibers decrease with an increase of PLLA content at the same conversion rate. However, the influence of the PLLA domain seems to weaken in the middle and later stages of decomposition reaction, indicating that the thermal degradation of the POM domain dominates the whole decomposition reaction. According to the Kissinger’ model [62], the peak temperatures given by the maxima of the first derivative weight-loss thermogram can be used to calculate the apparent activation energy of thermal decomposition reaction using the following equation:

(12) $$\ln\left(\frac{\beta }{T_{\max}^{2}}\right)=\ln\left(\frac{A\cdot R}{\Delta E_{a}}\right)-\frac{\Delta E_{a}}{R}\cdot \frac{1}{T}$$

where *β* is the heating rate, *T* the absolute temperature corresponding to the conversion, Δ*E*_a_ the apparent activation energy, and *R* is the gas constant. On the other hand, a new equation can be developed by Flynn and Wall to calculate the decomposition kinetic parameters of fiber samples and is expressed as [63]:

(13) $$\ln\beta =\ln\left(\frac{Z\cdot \Delta E_{a}}{R}\right)-\ln\alpha -0.4567\frac{\Delta E_{a}}{R}\cdot \frac{1}{T}.$$

Based on the Flynn–Wall’s model, the apparent activation energy of thermal decomposition reaction for the pure POM fiber and POM/PLLA bicomponent fibers can be determined by the slope of the linear plot of ln*β* versus 1/*T* at a fixed conversion (normally taken as 5% conversion) [63]. Figure S3 shows the Flynn–Wall plots of ln*β* versus 1/*T* at the conversion rate of 5% for all of fiber samples, and the obtained data of Δ*E*_a_ are given in Table S3. Pure POM fiber is found to have a high Δ*E*_a_ value of 384.6 kJ/mol for the thermal degradation reaction at a conversion rate of 5%. It is important to note that the Δ*E*_a_ value of the bicomponent fibers tends to decrease with the addition of PLLA into POM, and the higher content of PLLA brings about lower apparent activation energy. It is well known that the apparent activation energy is associated with the initial decomposition stage [64]. According to our previous study, pure PLLA has a much lower Δ*E*_a_ value of 309.2 kJ/mol compared to pure POM [35], implicating that there is a lower energy barrier for PLLA to perform the thermal decomposition reaction and therefore the PLLA domain degrades earlier than the POM one. These decomposition kinetic results clearly demonstrated that the incorporation of PLLA could initiate the thermal decomposition reaction more easily due to the reduction of energy barrier.

### 3.8. Lifetime Predication

The determination of the serve lifetime of POM/PLLA bicomponent fibers is very important for their successful applications for engineering and consumer-goods areas. It is well known that the serve lifetimes of synthetic fibers are controlled by the chemical reactions resulting in chain scission as well as the mediating environmental factors. Therefore, through understanding the physical or chemical changes in the structure of synthetic fibers when exposed to aggressive environments, one can achieved a framework for controlling their ultimate service lifetimes by either stabilizing the polymer or chemically accelerating the degradation reactions [64]. To evaluation the lifetime of high-temperature polymers, D.J. Toop proposed a theory of thermal lifetime prediction by means of the TGA technique and established a connection between the TGA and long-term life testing. According to Toop’s model, a continuous kinetic function was first established in terms of single decomposition or rearrangement reaction for the thermal degradation of polymers, and then the temperature dependence of chemical reaction rate was given by the Arrhenius equation. In this case, the lifetime test can be conducted on the basis of the apparent activity energy obtained from the thermal decomposition kinetics by means of the Arrhenius equation using the following three simple equations:

(14) $$x_{f}=\Delta E_{a}/RT_{5\%}$$

(15) $$\log\left[p(x_{f})\right]=-2.315-0.457x_{f}$$

(16) $$\log t_{f}=\frac{\Delta E_{a}}{RT_{f}}+\log\left(\frac{\Delta E_{a}}{\beta \cdot R}\cdot p(x_{f})\right)$$

where *x*_f_ is the variable set as Δ*E*_a_/*RT*_5%_, *T*_5%_ the absolute temperature at 5% weight loss, *T*_f_ the failure temperature, *t*_f_ the approximate time of failure, and log[p(*x*_f_)] is the linear function of *x*_f_. In this work, the thermal lifetimes of the pure POM fiber and POM/PLLA bicomponent fibers in the temperature range of 50–140 °C were predicted according to the values of apparent activity energy obtained from the Flynn-Wall’s model by used of Equations (14)–(16) [61,62].

**Figure 16:**
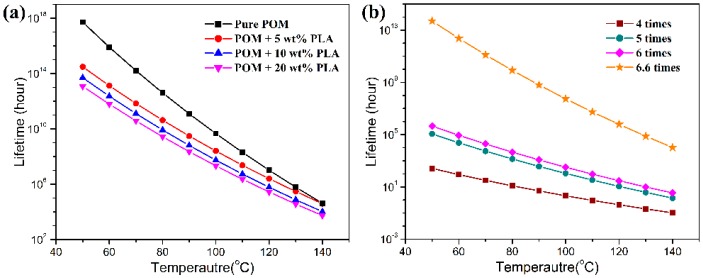
Plots of lifetime as a function of temperature for (**a**) pure POM fiber and POM/PLLA bicomponent fibers at the ultimate draw ratio, and (**b**) POM/PLLA bicomponent fiber containing 10 wt % PLLA at different draw ratios.

Figure 16 shows the thermal lifetimes of all the fiber samples at different temperatures and draw ratios on the basis of the decomposition kinetic data obtained from TGA measurements. It is expected that pure POM fiber presents a much longer lifetime than the bicomponent fibers in the temperature range between 50 and 140 °C due to its high degree of crystallinity and good structural stability. Moreover, there is a significant effect of temperature on the lifetime of the POM fiber, and it is evidently understandable that the higher ambient temperature results in a shorter lifetime due to the effect of thermal stability. As seen in Figure 16a, the bicomponent fibers are observed to exhibit a downward trend in lifetime in the presence of PLLA at all ambient temperatures. The bicomponent fibers are predicted to have the lifetimes of approximately 3.0 × 10^14^, 5.0 × 10^13^, and 1.2 × 10^13^ hours at the PLLA contents of 5, 10, and 20 wt %, respectively. These data are obviously lower than the lifespan of 5.15 × 10^17^ hours for pure POM fibers. Furthermore, the lifetime tends to decrease with an increase of PLLA content, which may be ascribed to the poorer thermal stability of the PLLA phase in the bicomponent fibers. It is noteworthy that the lifetimes of the bicomponent fiber samples tend to be close to each other with an increase of ambient temperatures, indicating that the POM domain dominates the thermal lifetime at high temperatures. In addition, this work also conducted an investigation on the effect of the draw ratio on the lifetime of the bicomponent fibers containing 10 wt % PLLA as a representative sample, and the obtained results are presented in Figure 16b. It is interesting to note that the lifetime of the bicomponent fibers is greatly influenced by draw ratio, and the higher the draw ratio, the longer the lifetime. This phenomenon can be explained by the fact that the higher draw ratio leads to a higher degree of crystallinity and more stable crystallites at a higher orientation level. As a result, the thermal stability of the bicomponent fibers is improved at the high draw ratio. There is no doubt that the introduction of the appropriate percentage of PLLA not only facilitates the natural degradation of POM/PLLA bicomponent fibers but also reserve satisfactory mechanical performance for the bicomponent fibers within their serve lifetimes.

## 4. Conclusions

In this investigation, the spinnability of POM/PLLA blends was first confirmed by the rheological evaluation and SSA thermal fractionation analysis, and then the POM/PLLA bicomponent fibers were successfully melt spun by using POM/PLLA blends, followed by hot drawing. The morphological observation indicated that the prepared bicomponent fibers achieved a smooth surface, uniform diameter distribution, and compact texture at the ultimate draw ratios, and the incorporation of PLLA did not influence their external profile, internal structure and diameter. In the resulting bicomponent fibers, the POM domain showed a decrease of the degree of crystallinity in the presence of the amorphous PLLA phase, however, its crystallinity was enhanced with an increase of draw ratio due to the stress-induced crystallization effect. The post drawing not only led to the crystalline orientation of lamellar folded-chain crystallites for the POM phase but also promoted the macromolecular rearrangement of two domains. As a result, the bicomponent fibers containing 5 and 10 wt % PLLA achieved the relatively high tensile strength of 791 and 742 MPa at the ultimate draw ratio, respectively. The bicomponent fibers gained a partial hydration capability in both acid and alkali media and therefore could meet the requirement for serving as a type of biodegradable fibers. The incorporation of the appropriate percentage of PLLA into POM slightly reduced the thermo-oxidative aging property and thermal stability of the bicomponent fibers. Such a combination of two polymers shortens the thermal lifetime of the bicomponent fibers but facilitates their natural degradation for ecological and sustainable applications.

## Supplementary Materials

The following are available online at https://www.mdpi.com/2073-4360/11/11/1753/s1, Video S1: Melt-spinning and post-drawing procedure for POM/PLLA bicomponent fibers; Figure S1: Plot of MFR of POM/PLLA blends as a function of PLLA content; Figure S2: Numerically deconvolved DSC thermograms of POM/PLLA bicomponent fibers; Figure S3: Flynn-Wall plots of ln*β* versus l/*T*_max_ for the thermal degradation reaction of pure POM fiber and POM/PLLA bicomponent fibers at the ultimate draw ratios; Table S1: The thermal degradation temperatures as a function of percentage conversion for pure POM, PLA and POM/PLA blends at different heating rates; Table S2: Thermo-oxidation coefficients (*K*_TO_) of pure POM fiber and POM/PLLA bicomponent fibers at different draw ratios; Table S3: Thermal degradation kinetic data obtained from TGA measurements for pure POM fiber and POM/PLLA bicomponent fibers; Table S4: Thermal degradation temperatures as a function of conversion rate for pure POM fiber and POM/PLLA bicomponent fibers at different heating rates.
