# Supplementary material for: Crystalline Characteristics, Mechanical Properties, Thermal Degradation Kinetics and Hydration Behavior of Biodegradable Fibers Melt-Spun from Polyoxymethylene/Poly(l-lactic acid) Blends
Source: Polymers (Basel). 2019 Oct 25;11(11):1753. doi: 10.3390/polym11111753 (PMC6918227; doi:10.3390/polym11111753)
Supplement: Supplementary file 1 [file polymers-11-01753-s001.zip › Supplementary Materials.doc]

**Supplementary Materials**

Crystalline Characteristics, Mechanical Properties, Thermal Degradation Kinetics and Hydration Behavior of Biodegradable Fibers Melt-Spun from Polyoxymethylene/Poly(l-lactic acid) Blends

Jianhua Li ^1,2^, Yatao Wang ^2^, Xiaodong Wang ^1,^* and Dezhen Wu ^1,^*

^1^	State Key Laboratory of Organic–Inorganic Composites, Beijing University of Chemical Technology, Beijing 100029, China

^2^	Coal Chemical R & D Center, Kailuan Group Limited Liability Corporation, Tangshan, Hebei 063018, China

*	Correspondence: wangxdfox@aliyun.com (X.W.); wdz@mail.buct.edu.cn (D.W.); Tel.: +86-10-6442-1693 (X.W.)

**Figure S1.** Plot of MFR of POM/PLLA blends as a function of PLLA content.

**Figure S2.** Numerically deconvolved DSC thermograms of POM/PLLA bicomponent fibers containing (a) 5, (b) 10 and (c) 20 wt % PLLA at different draw ratios.

**Figure S3.** Flynn-Wall plots of ln*β* versus l/T_max_ for the thermal degradation reaction of pure POM fiber and POM/PLLA bicomponent fibers at the ultimate draw ratios.

**Table S1.** Thermal degradation temperatures as a function of percentage conversion for pure POM, PLA and POM/PLA blends at different heating rates.

| Blend Sample | *T*_m,1_  (°C) | *n*_1_  (%) | *T*_m,2_  (°C) | *n*_2_  (%) | *T*_m,3_  (°C) | *n*_3_  (%) | *T*_m,4_  (°C) | *n*_4_  (%) | *T*_m,5_  (°C) | *n*_5_  (%) | *T*_m,6_  (°C) | *n*_6_  (%) | *T*_m,7_  (°C) | *n*_7_  (%) | *T*_m,8_  (°C) | *n*_8_  (%) | *T*_m,9_  (°C) | *n*_9_  (%) |
| --- | --- | --- | --- | --- | --- | --- | --- | --- | --- | --- | --- | --- | --- | --- | --- | --- | --- | --- |
| Pure POM | 167.6 | 28.6 | 166.9 | 31.6 | 165.0 | 17.8 | 162.7 | 5.8 | 160.5 | 3.1 | 157.7 | 2.5 | 152.7 | 4.8 | 146.6 | 5.9 | – | – |
| POM + 5 wt % PLA | 167.9 | 32.5 | 166.8 | 24.3 | 165.2 | 22.4 | 162.3 | 4.7 | 160.9 | 4.4 | 157.8 | 2.7 | 152.3 | 5.2 | 146.7 | 3.8 | – | – |
| POM + 10 wt % PLA | 167.8 | 36.3 | 166.8 | 16.4 | 165.3 | 24.8 | 162.6 | 5.2 | 160.5 | 5.5 | 157.8 | 2.9 | 151.9 | 6.1 | 146.7 | 2.9 | – | – |
| POM + 20 wt % PLA | 167.9 | 53.7 | 166.8 | 9.7 | 165.5 | 16.3 | 163.1 | 4.8 | 161.2 | 3.0 | 159.0 | 2.9 | 152.8 | 4.7 | 147.9 | 2.5 | 171.6 | 2.3 |

**Table S2.** Thermo-oxidation coefficients (*K*_TO_) of pure POM fiber and POM/PLLA bicomponent fibers at different draw ratios.

| Fiber sample | Draw ratio (times) | *K*_TO_ according to tensile strength | *K*_TO_ according to elongation at break |
| --- | --- | --- | --- |
| Pure POM | 4 | 0.99 | 0.99 |
|  | 5 | 0.99 | 0.98 |
|  | 6 | 0.99 | 0.97 |
|  | 6.6 | 0.98 | 0.97 |
| POM+5 wt % PLA | 4 | 0.98 | 0.96 |
|  | 5 | 0.96 | 0.95 |
|  | 6 | 0.95 | 0.94 |
|  | 6.6 | 0.92 | 0.93 |
| POM+10 wt % PLA | 4 | 0.95 | 0.93 |
|  | 5 | 0.92 | 0.90 |
|  | 6 | 0.90 | 0.91 |
|  | 6.6 | 0.85 | 0.87 |
| POM+20 wt % PLA | 4 | 0.93 | 0.92 |
|  | 5 | 0.91 | 0.90 |
|  | 6 | 0.89 | 0.87 |

**Table S3.** Thermal degradation kinetic data obtained from TGA measurements for pure POM fiber and POM/PLLA bicomponent fibers.

| Fiber sample | *β*  (°C/min) | *T*_i_  (°C) | *T* _max,1_  (°C) | *α*_max,1_  (%/min) | *T*_max,2_  (°C) | *α*_max,2_  (%/min) | *T*_f_  (°C) | ∆*E*_a_  (KJ/mol) |
| --- | --- | --- | --- | --- | --- | --- | --- | --- |
| Pure POM | 5 | 284.6 | – | – | 358.9 | 6.3 | 404.9 | 384.6 |
|  | 10 | 288.9 | – | – | 384.6 | 10.4 | 425.3 |  |
|  | 15 | 301.7 | – | – | 396.5 | 15.3 | 437.5 |  |
|  | 20 | 302.9 | – | – | 397.7 | 19.2 | 443.6 |  |
| POM + 5 wt % PLA | 5 | 245.2 | 337.3 | 3.7 | 383.6 | 8.0 | 399.1 | 280.9 |
|  | 10 | 279.1 | 337.5 | 4.3% | 402.7 | 16.0 | 414.6 |  |
|  | 15 | 280.4 | 361.0 | 10.5 | 405.1 | 20.9 | 421.4 |  |
|  | 20 | 286.6 | 364.5 | 14.8 | 405.5 | 23.2 | 425.4 |  |
| POM + 10 wt % PLA | 5 | 252.9 | 313.0 | 2.2 | 383.9 | 8.1 | 399.4 | 274.3 |
|  | 10 | 265.9 | 330.6 | 4.9 | 399.6 | 15.9 | 415.1 |  |
|  | 15 | 255.5 | 357.2 | 5.9 | 408.2 | 24.9 | 426.7 |  |
|  | 20 | 259.4 | 349.9 | 8.2 | 408.8 | 29.5 | 427.9 |  |
| POM + 20 wt % PLA | 5 | 254.8 | 296.4 | 4.0 | 386.9 | 7.6 | 393.3 | 265.5 |
|  | 10 | 278.6 | 321.6 | 8.9 | 391.4 | 14.8 | 405.2 |  |
|  | 15 | 254.1 | 285.2 | 6.0 | 405.5 | 23.9 | 423.8 |  |
|  | 20 | 274.5 | 305.5 | 9.5 | 409.6 | 26.3 | 428.0 |  |

**Table S4.** Thermal degradation temperatures as a function of conversion rate for pure POM fiber and POM/PLLA bicomponent fibers at different heating rates.

| β  (°C/min) | Conversion rate  (%) | Thermal degradation temperature (°C) | | | |
| --- | --- | --- | --- | --- | --- |
|  |  | Pure POM | POM+5 wt % PLA | POM+10 wt % PLA | POM+20 wt % PLA |
| 5 | 5 | 284.6 | 245.2 | 252.9 | 254.8 |
|  | 10 | 308.2 | 284.4 | 274.9 | 275.1 |
|  | 20 | 327.2 | 326.7 | 301.5 | 294.4 |
|  | 30 | 339.7 | 342.2 | 326.0 | 308.1 |
|  | 40 | 349.1 | 355.8 | 352.9 | 325.2 |
|  | 50 | 356.5 | 366.9 | 367.2 | 351.8 |
|  | 60 | 363.7 | 374.7 | 375.5 | 367.2 |
|  | 70 | 372.5 | 381.3 | 382.3 | 375.7 |
|  | 80 | 384.3 | 387.5 | 388.6 | 382.6 |
|  | 90 | 397.3 | 394.7 | 395.3 | 389.4 |
| 10 | 5 | 288.9 | 279.1 | 265.9 | 278.6 |
|  | 10 | 312.5 | 303.1 | 289.0 | 296.2 |
|  | 20 | 334.2 | 333.3 | 319.6 | 312.2 |
|  | 30 | 351.3 | 353.9 | 341.9 | 323.5 |
|  | 40 | 364.7 | 369.7 | 365.9 | 335.5 |
|  | 50 | 376.2 | 381.4 | 380.2 | 354.4 |
|  | 60 | 387.6 | 389.5 | 389.3 | 376.8 |
|  | 70 | 398.2 | 395.8 | 396.2 | 385.4 |
|  | 80 | 408.1 | 402.5 | 402.7 | 392.5 |
|  | 90 | 418.3 | 409.8 | 410.1 | 400.3 |
| 15 | 5 | 301.7 | 280.4 | 255.5 | 254.1 |
|  | 10 | 325.1 | 308.9 | 280.8 | 273.3 |
|  | 20 | 346.1 | 340.2 | 334.6 | 299.0 |
|  | 30 | 362.1 | 356.8 | 363.8 | 333.6 |
|  | 40 | 376.1 | 371.6 | 384.7 | 371.5 |
|  | 50 | 388.6 | 383.4 | 394.6 | 389.7 |
|  | 60 | 400.0 | 392.9 | 401.6 | 398.1 |
|  | 70 | 410.3 | 400.6 | 407.9 | 404.5 |
|  | 80 | 419.9 | 408.3 | 414.4 | 410.9 |
|  | 90 | 430.6 | 416.4 | 421.6 | 418.1 |
| 20 | 5 | 302.9 | 286.6 | 259.5 | 274.5 |
|  | 10 | 325.9 | 313.3 | 287.5 | 292.9 |
|  | 20 | 349.3 | 341.2 | 339.4 | 312.6 |
|  | 30 | 365.5 | 357.1 | 365.5 | 334.6 |
|  | 40 | 379.5 | 369.5 | 383.3 | 361.0 |
|  | 50 | 392.5 | 381.6 | 396.2 | 388.1 |
|  | 60 | 404.7 | 392.9 | 403.2 | 400.4 |
|  | 70 | 415.9 | 402.2 | 410.1 | 408.3 |
|  | 80 | 425.4 | 410.8 | 416.7 | 415.2 |
|  | 90 | 436.3 | 419.4 | 423.9 | 422.4 |
